# Devitalisation of pathogens in stored pig slurry and potential risk related to its application to agricultural soil

**DOI:** 10.1007/s11356-017-0557-2

**Published:** 2017-10-31

**Authors:** Jan Venglovsky, Nada Sasakova, Gabriela Gregova, Ingrid Papajova, Frantisek Toth, Tatiana Szaboova

**Affiliations:** 1 University of Veterinary Medicine and Pharmacy in Kosice, Komenského 73, 041 81 Košice, Slovakia; 2 Institute of Parasitology of the Slovak Academy of Sciences, Hlinkova 3, 040 01 Košice, Slovakia

**Keywords:** Pig slurry, *E. coli*, *Salmonella typhimurium*, Devitalisation, Parasites

## Abstract

The study investigated the risks arising from application of pig slurry to soil in relation to viability of *Salmonella typhimurium, Escherichia coli,* total coliforms*,* faecal enterococci and eggs of *Ascaris suum* at different temperatures*.* Potential effect of changes in physico-chemical parameters, particularly dry matter (DM), pH and ammonia, were also investigated. Examination showed that *S. typhimurium* was devitalised after storage in the slurry for 115 days at 4 °C and after 90 days at 20 and 42 °C. Devitalization of *E. coli* and faecal entrerococci required more than 115 at temperature of 4 °C and faecal enterococci were recovered from slurry after 115 days of storage even at temperature of 20 °C. Total coliforms survived for 115 days at all investigated temperatures. Complete devitalization of *A. suum* eggs was not achieved even after 115 days at 42 °C. Our investigations indicated potential microbial and parasitic risk related to application of pig slurry to soil even after 115 days of storage.

## Introduction

### Microbiological and parasitical risks related to application of pig slurry to soil

Pig slurry is classified as a natural fertiliser of animal origin, being a mixture of faeces, urine, remains of fodder and water used for the elimination of faeces. It is generated in litterless housing systems, where animals are kept on slatted or partially slatted floors. In the aspect of chemical composition, slurry is not a uniform material.

Chemical composition varies as it depends on many factors, such as the type and the age of animals, their feeding system and maintenance, the quality of fodder, as well as the dilution of the slurry and its storage method (Marszalek et al. 2014).

There are considerable variations microbiological compositions. Slurry is characterised by a high level of bacterial population; it can contain saprophytic microorganisms, pathogenic bacteria, viruses and fungi, as well as eggs and oocysts of gastro-intestinal parasites (Cotta et al. 2003). In slurry, there can be micro-organisms excreted by animals together with faeces, urine, milk, blood, purulent discharges, nasal and throat discharges, as well as discharges from vaginal tracts and amniotic fluid (Marszalek et al. 2014).

Animal manure is applied to soil with the aim to supply important nutrients to plants and to improve the soil structure. However, land-applied manure can represent a major reservoir of micro-organisms distributed across the landscape and available for loss to surface and ground water resources. While manure application can be managed to prevent much of the potential loss of pathogens and indicators (e.g., by managing application rates and forms, incorporation into the soil and use of buffers and setbacks between fields and waterways), poorly managed manure applications (e.g., application at excessive rates, onto saturated or frozen soils or without soil incorporation) can result in significant pathogen and indicator losses to surface and ground water.

When applying animal wastes to soil, we are concerned from the microbiological point of view with the presence of representatives of the family *Enterobacteriacea*, the majority of which have zoonotic character (such as *Salmonella* sp., *Escherichia coli*) and other germs *(Mycobacterium* sp.*, Enterococcus* sp.*, Streptococcus* sp., *Staphylococcus* sp.), which can be a threat to both farm animals and man.

Pathogens can survive or even grow in manure as this substrate provides the necessary nutrients and protects them against UV radiation, desiccation, and elevated temperature. On the other hand, there are several factors which support their die-off, such as predation, competition and inorganic ammonia. Temperature in particular is a critical factor in pathogen survival, with cooler temperatures generally enabling longer survival times. Bacterial pathogens such as *Salmonella* and *E. coli* O157:H7 can survive for several months in manure when environmental conditions are favourable (low temperatures, good moisture level). Increased temperatures, on the other hand, hasten die-off. The extent of this effect varies by organism, but survival in manure generally drops markedly at temperatures exceeding 20 to 30 °C compared with survival at cool temperatures (1 to 9 °C) (Rogers and Haines 2005).

This dependence of survival times on temperature results in seasonal trends; for example, a study of *Salmonella typhimurium* in swine slurry showed survival times of 26 days during summer and 85 days during winter (Venglovsky et al. 2006).

Most pathogenic bacteria are present in the environment only sporadically, at very low levels, and are difficult and expensive to detect directly. This is the reason why monitoring of risks was frequently based on determination of presence of more numerous and easier to monitor indicators of faecal contamination, such as faecal coliforms, *E. coli* and enterococci. *Clostridium perfringens* and coliphages can also be used as prospective indicators due to their presence in manure from all animals (Perdek et al. 2003).

Another group that was frequently used as an indicator of recent faecal contamination were faecal enterococci, a group of species of the genus *Eterococcus*, such as *E. faecalis, E. faecium, E. avium, E. bovis, E. gallinarum* and *Str. equinus.*

However, Ottoson et al. (2013) were evaluated enterococci as indicators for *Salmonella* but significantly slower decay rate and different behaviour in the material made them unsuitable as indicators for *Salmonella* in manure disinfected by ammonia or urea. According to Reissbrodt et al. (2000) they can persist in the environment, e.g., in soil, water or sewage, depending on the long-term survival of heavily stressed cells, particularly the so-called viable-but-nonculturable (VNC) organisms that have limited ability to grow on conventional laboratory plating media but are capable of reviving under in vivo conditions and causing diseases (Brandl 2006).

Côté and Quessy (2005) analysed samples of liquid hog manure, soil and vegetable (washed and unwashed) for the presence of *Salmonella* and *E. coli*. After application of manure to soil, *E. coli* counts decreased exponentially in the surface layer (20 cm). The estimated average time required to reach undetectable concentrations of *E. coli* in sandy loam varied from 56 to 70 days, while in loamy sand, *E. coli* could not be recovered after 77 days. The maximal *Salmonella* persistence in soil was 54 days. *E. coli* and *Salmonella* were not detected in any vegetable samples.

Stocker et al. (2015) investigated depth-dependent survival of *E. coli* and enterococci in soil after manure application and simulated rainfall. *E. coli* concentrations initially increased and then slowly decreased, whereas populations of enterococci began to decrease from the start and were not detectable after 4 weeks in all cases, except for treatments that received the least intense rainfall application.

Holley et al. (2006) added a six-strain cocktail of *Salmonella* serovars (*Agona, Hadar, Heidelberg, Montevideo, Oranienburg,* and *Typhimurium*) to about 5 kg of two soils (to yield 5 log CFU/g) with different moisture content (60 ad or 80% of field capacity) and to fresh pig slurry which was latter added to two soils at 25 g/kg (to yield 5 log CFU/g *Salmonella*). Storage of these soil treatments for 180 days at temperature sequences representing winter to summer, spring to summer, summer to winter seasonal periods, with four different temperature step lasting 45 days. Results showed that the largest decreases occurred within the first week. Higher moisture of soil, addition of manure and clay soil increased *Salmonella* survival. All but one of the treatments indicated that a 30-day delay between the field application of manure in the spring or autumn and use of the land for growing crop would ensure reasonable minimization of crop and animal contamination by *Salmonella*.

It is now commonly accepted that fruit and vegetables can be involved in spreading of enteric infections. Recently reported outbreaks related to fresh products included cases of *Escherichia coli* O157:H7 (spinach, lettuce), *Salmonella typhimurium* and *S. newport* (tomatoes, lettuce), *S. thompson* (rocket) and hepatitis A (spring onion). Consumption of fresh vegetable products, for example, lettuce, spinach and tomatoes, are commonly considered a potential risk factor for infection with enteropathogens such as *Salmonella* and *Escherichia coli* O157. Routes of spreading of these pathogens include application of animal manure as fertiliser, irrigation with water contaminated with faeces, direct contamination by livestock, wild animals and birds and postharvest contamination related to processing and worker hygiene. At present, postharvest interventions play the most important role in limiting the number of enteropathogens present on fresh products. The effectiveness of sanitizers is an important factor but their use in organic production is limited, thus prevention of contamination should be a preferred strategy (Heaton and Jones 2008).

The persistence of pathogens in environmental media depends on environmental conditions and the survival characteristics of the microbes present. The most important factors affecting survival of pathogens include temperature, ultraviolet (UV) radiation, moisture, pH, availability of nutrients and competition for them concentration of ammonia in the medium and predation (Rogers and Haines 2005).

In soils, pathogen survival is influenced by temperature, moisture content, pH, predation, nutrient availability, competition with native soil microorganisms and organic matter content. Aside from temperature, moisture exerts an important control, with increased moisture promoting survival. Faecal coliform bacteria survive longer in organic soils than in mineral soils, possibly due to the greater capacity of organic soils to hold water (EPA-OW, 2013).

Excrements of farm animals are also a source of endoparasites (cysts, eggs, larvae of genera *Ascaris* sp., *Oesophagostomum* sp., *Trichuris* sp., *Strongyloides* sp., *Isospora* sp., *Eimeria* sp., *Giardia* sp., *Balantidium* sp. and others) that may cause massive parasitic infections in both specific hosts and non-specific ones, such as human. An important factor in spreading of endoparasitoses is high tenacity of some propagative stages of parasites (Papajova and Juris 2012). The parasitic propagative stages, mainly endoparasitic protozoa and helminths, develop mostly outside their host’s organism (Papajova and Juris, 2009). *Ascaris suum* eggs are hygienically the most problematic ones (Papajova and Juris, 2012). They are amongst the helminth eggs most resistant to environmental factors. They may survive in the nature for many years; therefore, they tend to accumulate in the environment (soil, water) (Crompton, 2001; Papajova and Juris, 2009; Olszewska et al., 2014).

*A. suum* infects pigs and besides, health consequences affects significantly economy of pig production due to production losses related to reduce efficiency of feed conversion and condemnation of “milk-spot” livers (Dubinský et al., 2000). The main route of *A. suum* eggs transmission is the transport and application of contaminated pig slurry directly to agricultural land or use of this slurry for spray irrigation after its previous dilution (Crompton, 2001; Venglovský et al., 2006; Olszewska et al., 2014).

## Materials and methods

Raw pig slurry obtained from a pig farm was used in the experiment. The slurry was stored for 115 days in closed plastic containers of volume 5 l as follows: (1) in a refrigerator at 4 °C; (2) in a thermostat at 20 °C; (3) in a thermostat at 42 °C. Examination was carried out in triplicate.

Before storage, lyophilised strain *S. typhimurium* SK 14/39 (SZÚ Prague, CR) was inoculated into the investigated slurry (initial count of *S. typhimurium* 3.6 × 10^9^ CFU. ml^−1^).

Plate counts of total coliforms and *E. coli* (CFU/ml) were determined on Endo agar (HiMedia, India) with incubation at 37 or 43 °C for 24 h, respectively.

Plate counts of faecal enterococci (CFU/ml) were determined on solid selective medium containing sodium azide (to suppress growth of Gram-negative bacteria) and colourless 2,3,5-trifenyltetrazolium chloride which is reduced by intestinal enterococci to red formazan. Typical colonies are convex, red, chestnut-brown or pink in colour either in the centre or throughout the colony surface.

Also, special polyurethane containers, each containing 1500 *A. suum* eggs, obtained by dissection of distal ends of the uterus of *A. suum* females, were introduced into the slurry.

Plate counts of tested bacterias (CFU/ml) were determined at days 0, 7, 12, 22, 32, 40, 55, 90 and 115 of storage. At the same intervals, devitalisation of *A.suum* eggs was observed in comparison with *A.suum* eggs stored in distilled water at the same temperatures.

The relevant physical and chemical properties were determined in homogenised slurry as follows: pH was determined by direct measurement with a glass electrode pH meter (HACH); dry mater (DM) was analysed by drying samples at 105 °C to a constant weight; ammonium ions (NH_4_^+^) were analysed by steam distillation and titration.

The count of selected micro-organisms was expressed as mean of log_10_CFU. ml^−1^ ± standard deviation. Significance of differences between experimental and control groups was determined using Student’s *t* test and ANOVA test at the levels of significance 0.05, 0.01 and 0.001 (Statistica 6.0).

The number of viable *A. suum* eggs was expressed as mean ± standard deviation. Significance of differences between experimental and control groups of parasites was determined using Student’s *t* test, ANOVA and Dunnet Multiple Comparison test at the levels of significance 0.05, 0.01 and 0.001 (Statistica 6.0).

The methods of regression analysis (least squares methods and the robust regression method) were used for the correlation between changes of physico-chemical parameters of stored slurry and survival of model organisms (bacteria and parasites).

## Results and discussion

Reduction of enteric pathogens in animal manures before field application is essential for mitigating the risk of foodborne illness associated with produce. The aim of the study was to investigate the viability of *S. typhimurium, E. coli,* total coliforms*,* faecal enterococci and *A. suum* eggs in raw pig slurry stored at different temperatures in order to assess the microbial and parasitic risks related to application of animal wastes to soil.

**Fig. 1:**
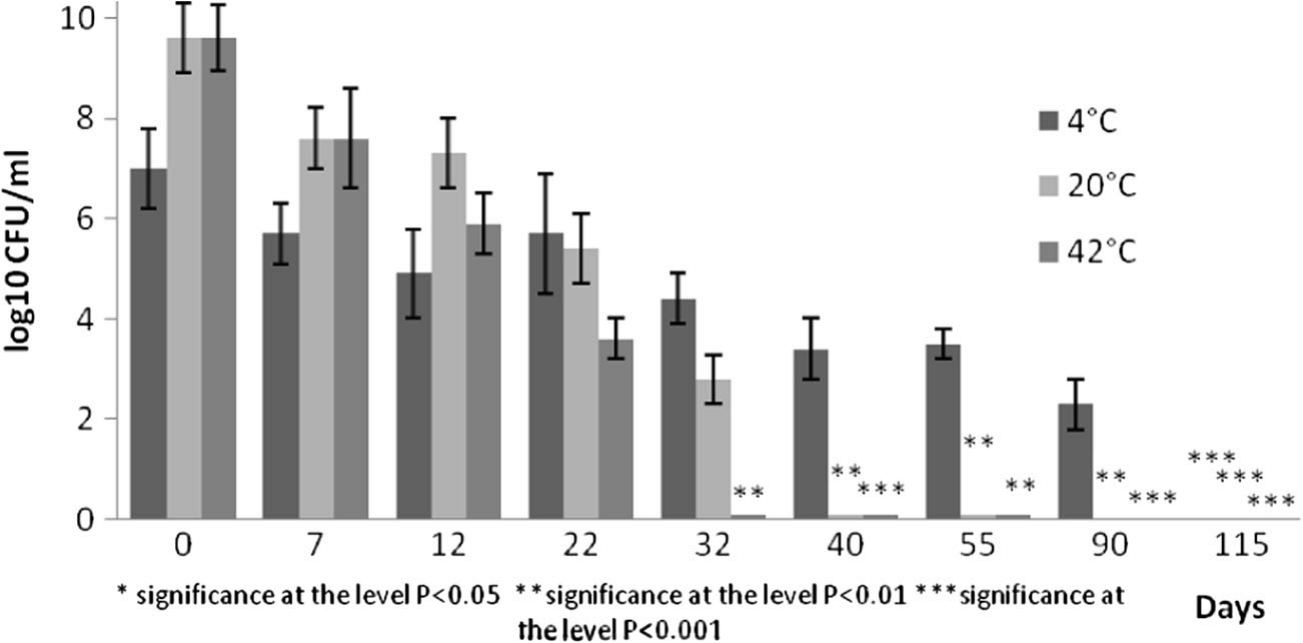
Survival of *S. typhimurium* at different temperatures expressed as log10 CFU ml^−1^

**Fig. 2:**
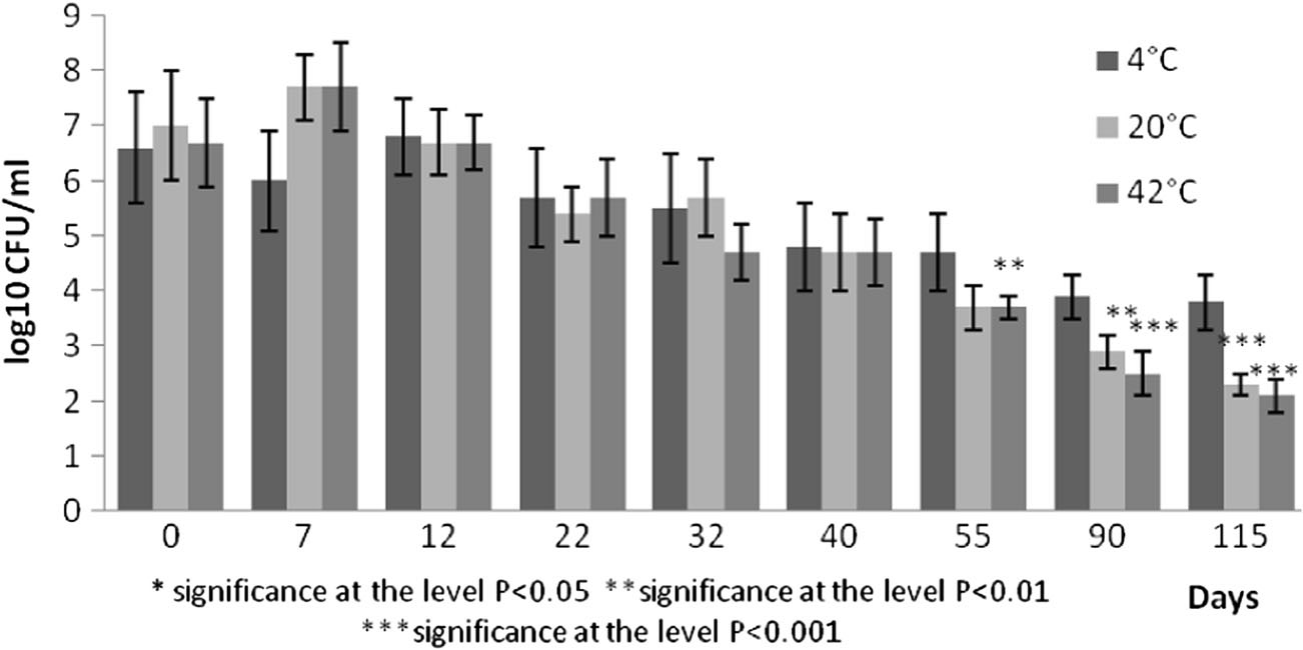
Survival of *Total coliforms* at different temperatures expressed as log10 CFU ml^−1^

**Fig. 3:**
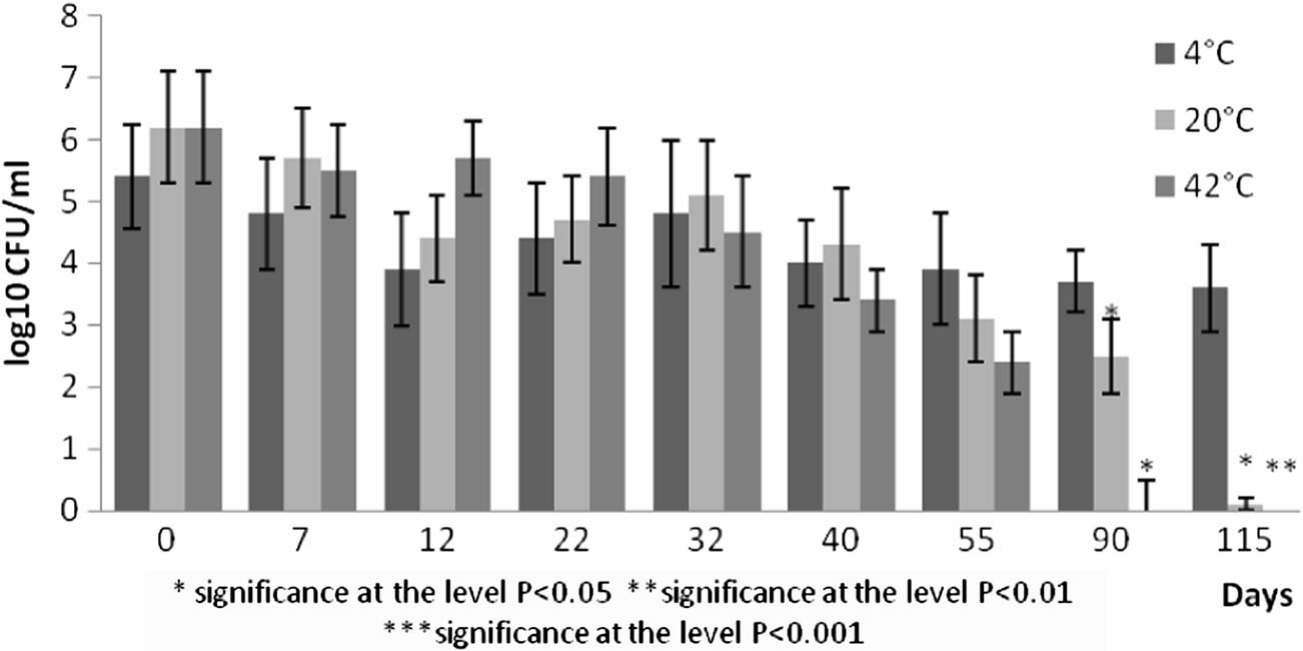
Survival of *E.coli* at different temperatures expressed as log10 CFU/ml

**Fig. 4:**
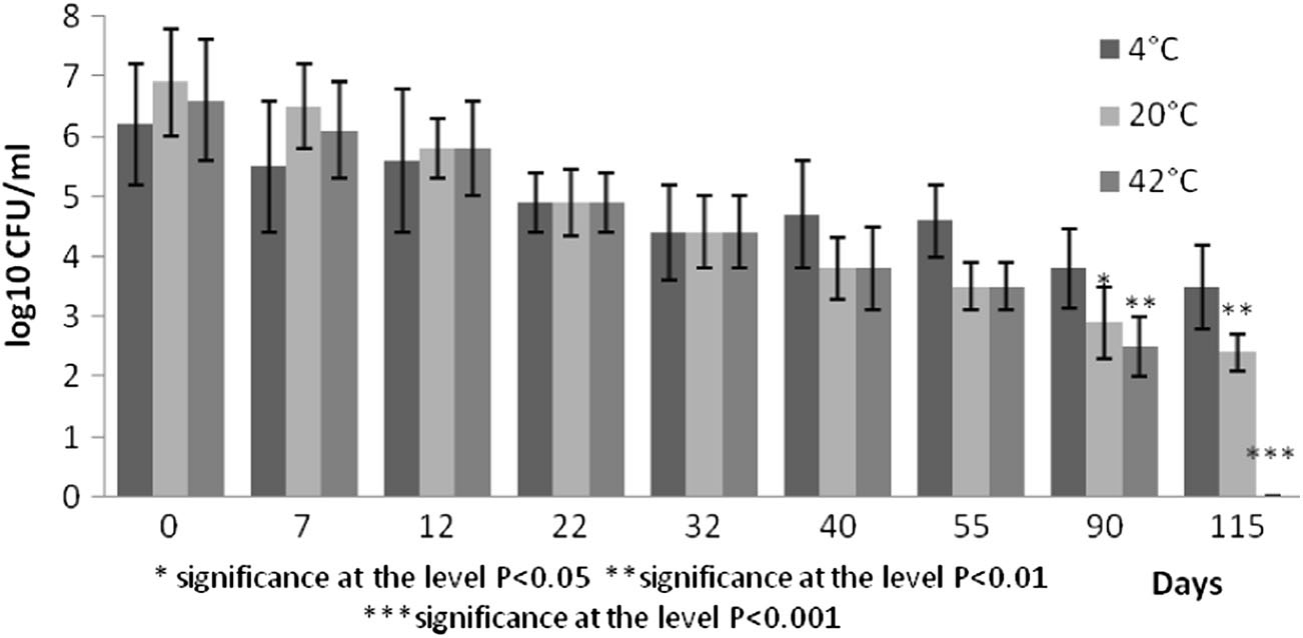
Survival of *Faecal enterococci* at different temperatures expressed as log10 CFU/ml

The plate counts of investigated micro-organisms in slurry stored at different temperatures are illustrated in Figs. 1, 2, 3 and 4. The initial concentration of the tested *S. typhimurium* strain in manure stored at 4 °C decreased by three orders of magnitude by day 90, and by day 115, the test strain was not recovered. Storage at 20 °C resulted in a marked decrease by seven orders of magnitude by day 32, and after this day, the test strain was detected only qualitatively. The most pronounced reduction in plate counts of *S. typhimurium* (*P* < 0.01) was observed after storage at 42 °C after 32 days exposure (Fig. 1).

Indicator bacteria represented by *E. coli* decreased by two orders at 4 °C and six orders at 20 °C by the day 115. Their counts decreased by six orders by the day 90 at 42 °C (Fig. 2). Abatement of total coliforms was by two orders (4 °C) and by four orders (20 °C, 42 °C). Similar decrease was observed for faecal enterococci decreased by two to three orders. The most marked decrease by six orders in plate counts of faecal enterococci was recorded in pig slurry stored at 42 °C in 115 days.

The effect of long-term storage (up to 185 days) of slurry on the survival of some indicator microorganisms was investigated under practical conditions. Whereas, the total bacterial counts as well as the numbers of *Enterobacteriaceae*, *E.coli* and faecal enterococci remained members of colony forming units of Salmonella exposed to the slurry by a special germ carrier technique including semipermeable membranes were reduced by more than five log10. *Yersinia colitica* was completely inactivated within only a few days, whereas the eggs of *Ascaris suum* lost their viability very slowly (Rapp, 1995).

Howell et al. (1996) observed that faecal coliforms experienced greater growth under warm conditions than faecal enterococcus. The authors of this study observed frequent regrowth of faecal coliforms but not of faecal enterococci.

Although survival of Salmonella in soil for more than 900 days was known earlier, its pathogenicity for roots and other plant parts remained questionable.

Incorporating wastes into the soil by tillage may enhance survival of micro-organisms because they are sheltered from lethal stresses, but incorporation also removes micro-organisms from interaction with surface runoff and places faecal microorganisms in an environment where predation by soil organisms can further reduce their numbers. Liquid manure-applied pasture is deposited on vegetation, where desiccation, high temperatures, and sunlight will devitalize majority of micro-organisms. However, faecal bacteria appear to survive for long periods (e.g., > 100 days) in cowpies, where they are relatively protected from the environmental factors and can eventually pass to soil. In the soil, micro-organisms are effectively removed from the percolating water by adsorption, filtration and predation (EPA-OW 2013).

Manure management methods should consider the different survival capabilities of the various pathogens and relevant properties of the manure that may affect their devitalisation. There are many unanswered questions in this area that warrant further research. Timing of manure land application is an important factor in minimising pathogen transport via runoff. Transport of microorganisms in runoff is more likely if excess manure is applied or if manure is misapplied (USEPA 2002).

Arrus et al. (2006) investigated the influence of temperature on survival of *S. typhimurium* and observed that did not grow in hog manure. However, storage reservoir temperatures facilitated salmonella survival during winter which contributed to the risk of contamination of fields at spring application (Reissbrodt et al. 2000).

Besides temperature and time of storage, the survival of pathogens in the slurry may well depend on factors other than temperature and duration of heat treatment, e.g., moisture content, free ammonia concentration, pH, the presence of other micro-organisms and other physico-chemical properties (Venglovský et al. 2006). This was the reason why the stored pig slurry was subjected also to physico-chemical examination.

**Fig. 5:**
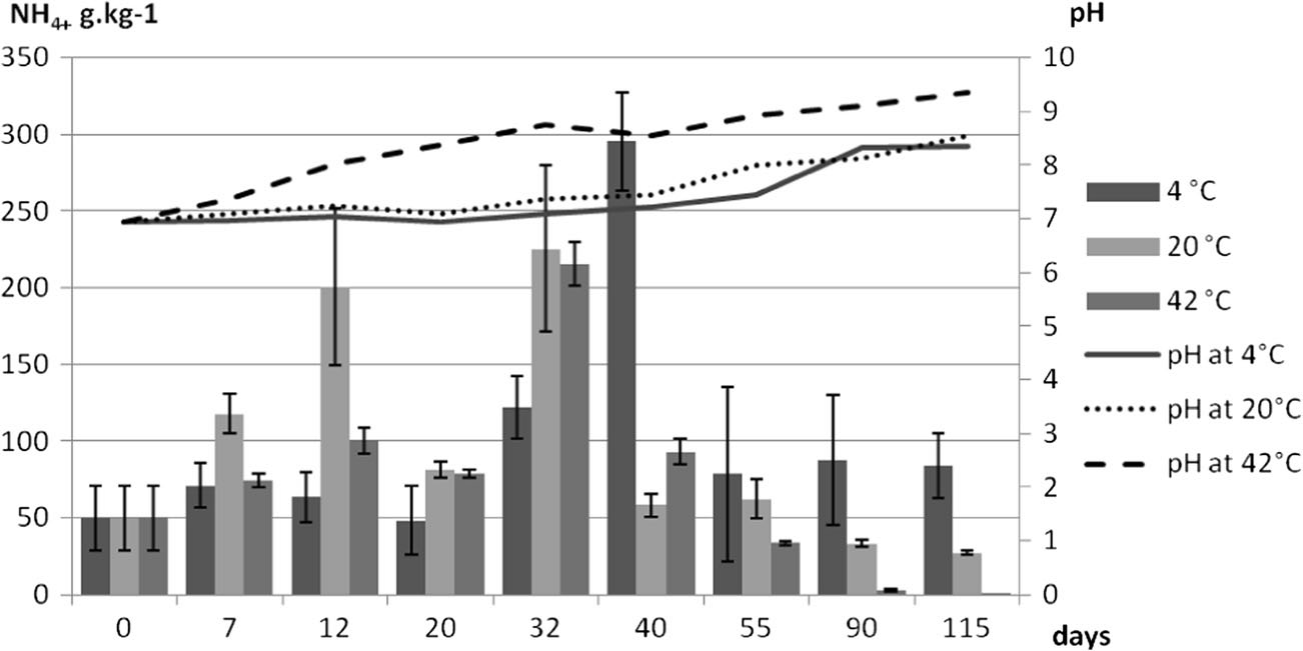
The course of pH and ammonium concentrations in slurry at different temperature

The initial DM content gradually decreased throughout the experiment from the 28.00 ± 6.26 g kg^−1^ to 15.09 ± 1.23 at 4 °C, 13.09 ± 4.12 at 20 °C and 12.89 ± 1.05 g kg^−1^ DM at 42 °C. This decrease was most likely related to the release of some volatile compounds from slurry as a result of decomposition processes taking place during its storage. The changes in pH and ammonia level in pig slurry stored at different temperatures are illustrated in Fig. 5.

With some fluctuations, the pH level increased throughout the storage at all three temperatures from the initial 6.95 ± 0.11 to 8.35 ± 0.58 at 4 °C, to 8.55 ± 0.17 at 20 °C and to 9.35 ± 0.05 at 42 °C. While at 4 and 20 °C, it took 90 and 55 days, resp. to reach the level of 8.00; at 42 °C, this level was reached already after 12 days. The initial level of NH_4_^+^ in the slurry was 50.00 ± 21.11 g kg^−1^ DM; it fluctuated considerably during the experiment, and the final level of this parameter differed between the treatments. At 4 °C, it peaked at day 40 (295.5 g kg^−1^ DM) and then decreased to 84.13 ± 21.23 g kg^−1^ DM. At 20 °C, there were two peaks at days 12 and 32 (200.45 ± 50.83 and 225.45 ± 54.23 g kg^−1^ DM) and the final concentration was 27.11 ± 1.06 g kg^−1^ DM. These two peaks coincided with a slight increase in pH. At 42 °C, there was one distinct peak at day 32 (215.13 ± 14.12 g kg^−1^ DM) after which the concentration of this parameter decreased abruptly and at day 115, we recorded only 0.56 ± 0.01 g kg^−1^ DM. The peak at day 32 was again reflected in increased pH level. The peaks were most likely related to decomposition of nitrogenous substances in the slurry and the final decreased levels to volatilisation in the form of NH_3_ at pH above.

Correlation and regression analysis confirmed significant relationship only between the number of micro-organisms in the slurry and pH. Relationship between devitalization of investigated micro-organisms and the ammonium content in the slurry was not detected.

However, the initial content of ammonia in fresh slurry (DM content approx. 2.8%) reached only 0.14% and at the highest peak, it increased for short time to about 0.3–0.4% at the maximum (depending on DM), which was probably not high enough to exert some influence on devitalization of micro-organisms.

It is generally known that some eggs, infectious larvae (L3), oocysts or sporocysts can survive for considerable time in the environment, frequently for several years, even under unfavourable conditions. The main subject of concern is highly resistant eggs of some parasitic nematodes such as *Ascaris* spp., *Trichuris* spp. and coccidial oocysts (Juris et al. 2000).

Parasite survival in animal manures may also be related to temperature, but the trends are not as pronounced as those reported for bacterial pathogens. This is likely due to their ability to form cysts and oocysts for protection from environmental pressures. According to Olson (2001), *A. suum* eggs are highly resistant to inactivation in faeces where they may remain infectious for years. A direct contact with infected animal and its faeces, contaminated environment or food chain (water, vegetables) are considered potential risk factors (Papajova and Juris 2012).

Roesel et al. (2017) observed prevalence of gastrointestinal parasites in pigs reared on small farms in Uganda. Altogether, they examined 932 pigs and detected developmental stages of endoparasites in 61.4% of examined animals. In the faeces of pigs, they found most frequently Strongylid eggs (57.1%) and eggs of *Metastrongylus* spp. (7.6%), *Ascaris suum* (5.9%), *Strongyloides ransomi* (4.2%) and *Trichuris suis* (3.4%). Coccidial oocysts were found in 40.7% of the examined animals.

**Fig. 6:**
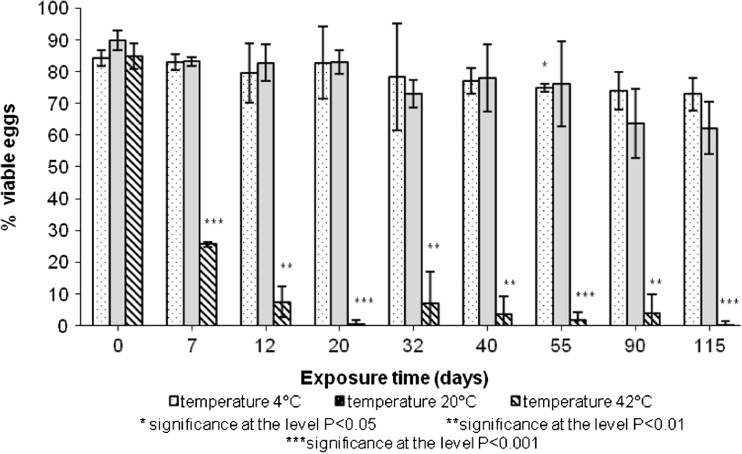
Viable *Ascaris suum* eggs in raw pig slurry

Our study showed that higher temperatures supported devitalisation of *A.suum* eggs (Fig. 6).

Results of statistical analysis (the least square methods and the robust regression method) indicated that the devitalisation of model organisms was most affected by temperature and pH. There was a quadratic relationship between the number of devitalised eggs and temperature while the relationship between the number of eggs and pH was linear. The effect of NH4+ concentration was insignificant.

Besides temperature and the time of storage, the survival of pathogens in the slurry may well depend on factors other than temperature and duration of heat treatment, e.g., moisture content, free ammonia concentration, pH, the presence of other micro-organisms and other physico-chemical properties (Venglovsky et al. 2006).

For this reason, the stored pig slurry was subjected also to physico-chemical examination. Changes in physico-chemical parameters were related to decomposition processes; however, some variations were observed which require further study.

## Conclusions

There are significant microbiological risks related to animal wastes spread onto land used for crop production or livestock grazing. Animal slurries are of particular concern, as the temperature in these substrates during their storage and some ways of common processing does not ensure complete devitalisation of potential bacterial and viral pathogens and eggs of parasites, as indicated by our observation of devitalisation of *S. typhimurium, E. coli,* total coliforms*,* faecal enterococci and *A. suum* eggs at 4, 20 and 42 °C during 115 days of storage.

In advanced countries, relevant legislative regulations require acceptable procedures for the disposal, processing and application of animal manures. However, there are still aspects that may raise some risk for safety of human food chain and require further investigations.

The best way is to put stress on preventive actions and measures that may eliminate any known or suspected danger resulting from pathogens present in animal manures applied to the soil that is used for animal grazing or growing of crops for human consumption.
